# Aptamer-Based Fluorometric Ochratoxin A Assay Based on Photoinduced Electron Transfer

**DOI:** 10.3390/toxins11020065

**Published:** 2019-01-24

**Authors:** Han Zhao, Xinying Xiang, Mingjian Chen, Changbei Ma

**Affiliations:** School of Life Sciences, Central South University, Changsha 410013, China; 172511001@csu.edu.cn (H.Z.); bestxxy@126.com (X.X.); chenmingjian@csu.edu.cn (M.C.)

**Keywords:** fluorescence, quencher-free, ochratoxin A, photoinduced electron transfer

## Abstract

This study describes a novel quencher-free fluorescent method for ochratoxin A (OTA) detection based on the photoinduced electron transfer (PIET) between guanine and fluorophore. In the absence of OTA, carboxyfluorescein (FAM)-labeled aptamer can partly hybridize with the complementary strand of OTA aptamer (OTA-cAPT), which contains four guanines at its 3′-end. As a result, the fluorescence of FAM is quenched due to PIET and stacked guanines. In the presence of OTA, FAM-labeled OTA aptamer can bind specifically to OTA, and thereby the high fluorescence intensity of the dye can be maintained. Under the optimal conditions, the method had a detection limit of 1.3 nM. In addition, the method we proposed is highly sensitive and specific for OTA. Furthermore, the method was proven to be reliable based on its successful application in the detection of OTA in red wine samples. Therefore, this promising, facile, and quencher-free method may be applied to detect other toxins by using other appropriate aptamers.

## 1. Introduction

Discovered in 1965, Ochratoxin A (OTA), a secondary metabolite in fungi, is one of the most common mycotoxins primarily produced by *Aspergillus ochraceus*, *Aspergillus carbonarius*, and *Penicillium verrucosum* (FAO/WHO, 2001) [1]. Food commodities, such as grains, beans, nuts, dried fruits, spices, wine, coffee, and meat products, are vulnerable to OTA contamination [2,3]. OTA can restrain protein synthesis, affect saccharide and calcium metabolisms, increase the rate of lipid of peroxidation, and disrupt the mitochondrial functions [4,5]. In order to prevent the potential risk resulting from OTA consumption, it is highly important to quantitatively analyze the level of OTA in contaminated raw materials. The determination of OTA has been carried out by a number of analytical methods, including thin layer chromatography (TLC), high-performance liquid chromatography (HPLC), mass spectrometry (MS), gas chromatography (GC), surface plasmon resonance (SPR), and so on [6,7,8,9,10]. Although these conventional methods have a number of advantages, such as high accuracy and selectivity, and low detection limits, they have many shortcomings, not only because they are time-consuming, but they also require high-priced instrumentation, sophisticated sample pretreatment, and trained personnel. In recent years, several economical and rapid immunoassays, including enzyme-linked immunosorbent assay (ELISA), have been employed to detect OTA [11,12,13]. Nevertheless, these methods are time-consuming and laborious. Hence, it can be beneficial to develop an alternative OTA detection method that is high-efficiency, simple, and low-cost.

Aptamers are single-stranded oligonucleotides or peptides that can be screened by an in vitro selection process called systematic evolution of ligands by exponential enrichment (SELEX). In this process, the aptamers bind with various target molecules, such as proteins, amino acids, tissues, small molecules, etc., with high affinity and specificity [14,15,16,17]. In comparison with antibodies, aptamers possess properties that can be easily chemically modified and higher stability, in addition to its lower cost and simpler synthesis process. The applications of aptamers can also be extended to analyze drugs and other biological molecules such as ATP, MicroRNA, glutathione, etc. [18,19,20,21]. Based on these advantages, a large number of aptamer-based methods have been applied to OTA detection [22,23,24]. For example, Jiang and Badie Bostan’s group team systematically introduced various strategies for OTA detection using aptasensors [25,26]. Lv’s group have developed a nuclease-aided target recycling signal amplification strategy for ochratoxin A monitoring [27]. Ma’s group used fluorometric aptamer-based determination of ochratoxin A based on the use of graphene oxide and RNase H-aided amplification [28]. 

Recently, methods for DNA and RNA analysis and metal ions detection based on the photoinduced electron transfer (PIET) between fluorophores and guanine bases has emerged [29,30]. As a result of PIET, the fluorescence of fluorophores can be quenched by the neighboring guanine molecules [31]. In this work, based on the high affinity between OTA and OTA aptamer, as well as the quenching ability of guanines, a simple and cost-effective quencher-free fluorescence method based on PIET was developed. The developed method was also successfully applied to detect OTA in red wine samples, and thus may have a promising application in the food safety fields.

## 2. Results and Discussion

### 2.1. Principle of OTA Detection

**Scheme 1:**
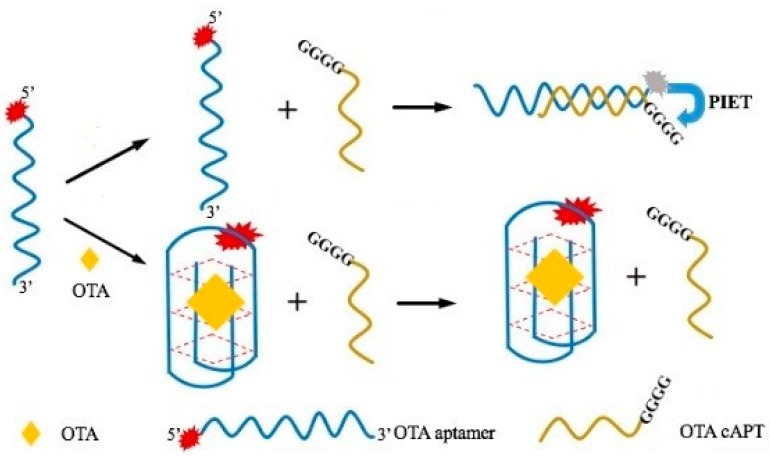
Schematic illustration of the fluorescent detection of ochratoxin A based on photoinduced electron transfer.

The principle of the proposed fluorescence assay in the detection of OTA is depicted in Scheme 1. The OTA aptamer sequence we used was firstly screened out by the team of Cruzaguado in 2008 [32]. OTA aptamer exhibits strong fluorescence intensity due to the fluorescein-based dye, FAM, attached to its 5′-end. OTA-cAPT partly hybridizes with its complementary OTA aptamer containing four guanines at its 3′-end, which are the fluorescence quenchers. In the absence of OTA, FAM-labeled OTA aptamer can hybridize with OTA-cAPT, forming double-stranded DNA. When FAM is in the proximity of the four guanines, the photoinduced electron transfer (PIET) between them is induced, thereby causing the fluorescence to be quenched. On the other hand, when OTA is present in the detection system, it can specifically bind FAM-labeled OTA aptamer with high affinity, folding into antiparallel G-quadruplex structure, which can protect fluorescence of the dye from being quenched by PIET. The change of fluorescence signal is closely linked with the concentration of OTA. By measuring the degree of fluorescence recovery, the amount of OTA can be determined.

### 2.2. Feasibility of the Proposed Method

**Figure 1:**
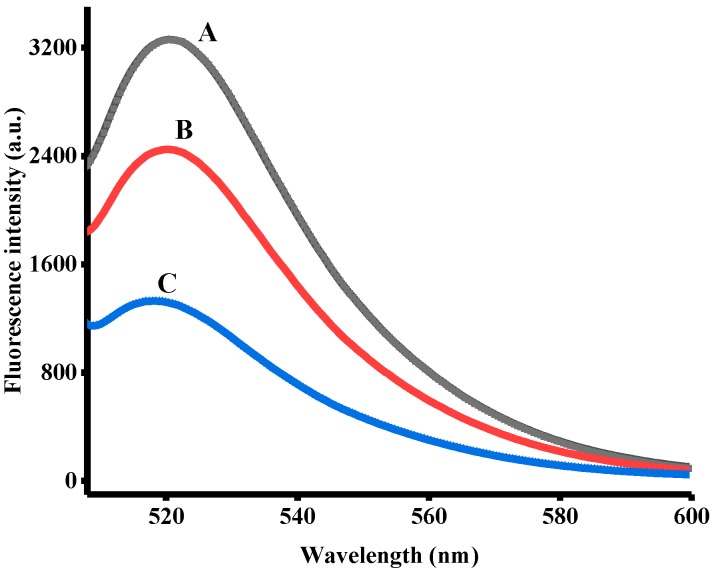
Fluorescence emission spectra for FAM-labeled ochratoxin A (OTA) aptamer (curve A) and reaction products in the presence (curve B) or the absence of OTA (curve C). The concentrations of FAM-labeled OTA aptamer, OTA cAPT and OTA were 200 nM, 400 nM and 2 μM respectively.

A series of experiments was carried out to evaluate the feasibility of the method. The fluorescence emission spectra of FAM-labeled OTA aptamer under different reaction conditions are shown in Figure 1. FAM-labeled OTA aptamer exhibited remarkable fluorescence intensity (curve A); However, the signal was rapidly decreased upon the addition of OTA-cAPT in the absence of OTA (curve C). It appears that the hybridization between FAM-labeled OTA aptamer and OTA-cAPT results in stacked guanines to be in the proximity of FAM dye, causing its fluorescence to be quenched due to PIET. However, as indicated by curve B (Figure 1), the fluorescence signal was recovered when OTA was present, due to the high affinity between OTA aptamer and OTA. This indicates that the G-quadruplex structure was formed, and could thereby maintain high fluorescence intensity of FAM. These data demonstrate that the proposed method can successfully be applied to detect OTA.

### 2.3. Optimization of Assay Conditions

**Figure 2:**
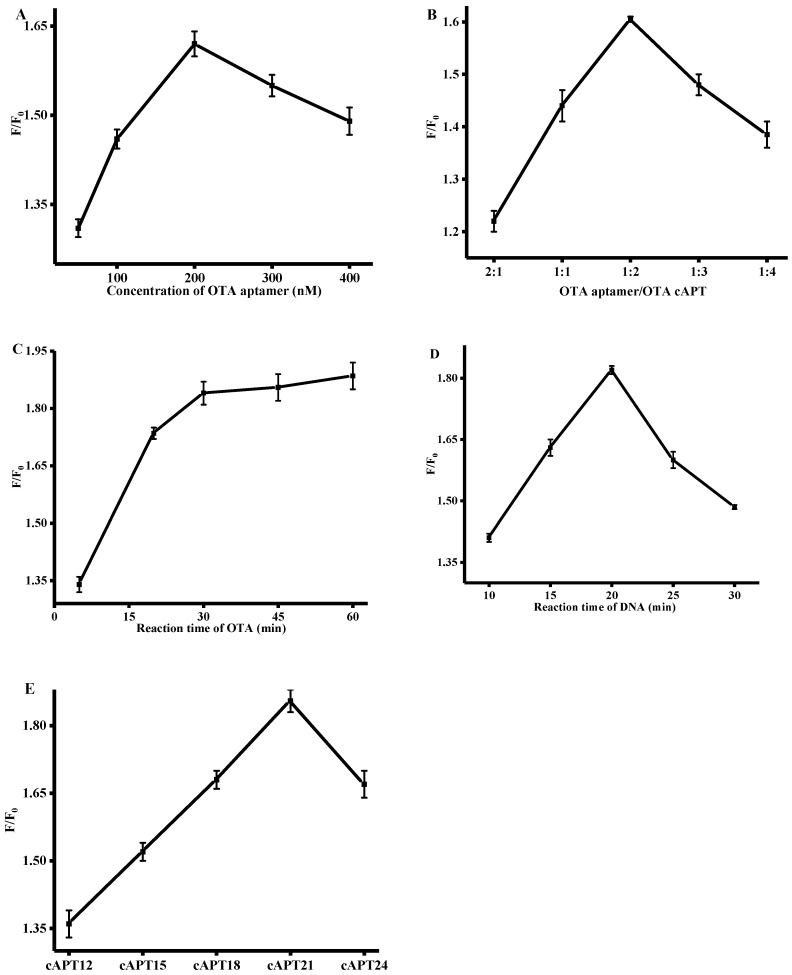
Optimization of ochratoxin A (OTA) detection conditions: (**A**) Concentration of OTA aptamer; (**B**) Concentration ratio of OTA aptamer to OTA cAPT; (**C**) reaction time for OTA; (**D**) reaction time for DNA probe; and (**E**) the length of OTA cAPT. F_0_ and F represent the fluorescence intensities in the absence and presence of OTA, respectively. Error bars were estimated from three replicate measurements.

It is critical to investigate parameters that may affect the fluorescence signal during OTA detection. The concentration range of OTA aptamer from 50 nM to 400 nM was first optimized. As shown in Figure 2A, the best concentration of OTA aptamer was 200 nM. The molar ratio of OTA aptamer to OTA-cAPT was then investigated, and as shown in Figure 2B, the optimal molar ratio was 1:2. The optimal concentrations of OTA aptamer and OTA-cAPT were 200 nM and 400 nM. As indicated in Figure 2C, the fluorescent intensity increased with increasing reaction time between OTA and OTA aptamer, reaching a plateau after 30 min, at which the hybridization between aptamers and OTA reaches equilibrium and saturation point. Thus, 30 min was chosen as the OTA incubation time. Furthermore, as can be observed in Figure 2D, 20 min was a suitable incubation time for OTA aptamer and OTA cAPT as well as for OTA detection. Finally, the effect of the length of OTA-cAPT on fluorescence signal was studied, and as indicated in Figure 2E, cAPT21 was selected throughout the experiments. 

### 2.4. Quantitative Detection of OTA

**Figure 3:**
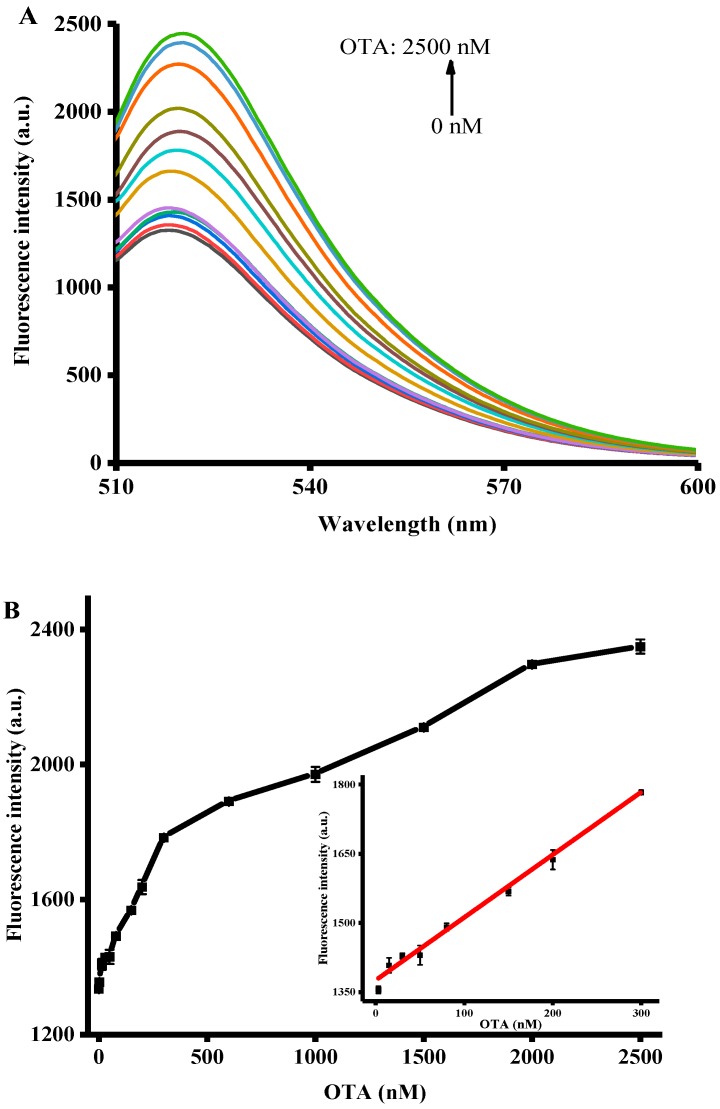
(**A**) Fluorescence spectra for ochratoxin A OTA at different concentrations. (**B**) Relationship between fluorescence intensity and OTA concentration. Error bars were estimated from three replicate measurements.

With the optimal conditions selected above, the detection of OTA in samples spiked with different concentrations of OTA was conducted. As shown in Figure 3A, the fluorescence intensity at 520 nm gradually increased with the increase of OTA concentration, from 0 to 2500 nM, and reached a plateau at 2000 nM. Figure 3B depicts the relationship between fluorescence intensity and OTA concentration. The inset of Figure 3B shows the calibration curve for fluorescence intensity versus OTA concentration. The curve has a good linear correlation at the concentration range of 3 to 300 nM. It has a linear regression equation of F = 1.361C + 1374.376 and a correlation coefficient of 0.99501 (where F represents the fluorescence intensity and C is the concentration of OTA). The calculated limit of detection for OTA was 1.3 nM, which is comparable to or better than those of other methods (Table 1). Therefore, we may conclude that the proposed assay can easily and successfully quantify OTA.

### 2.5. Selectivity of OTA Assay

**Figure 4:**
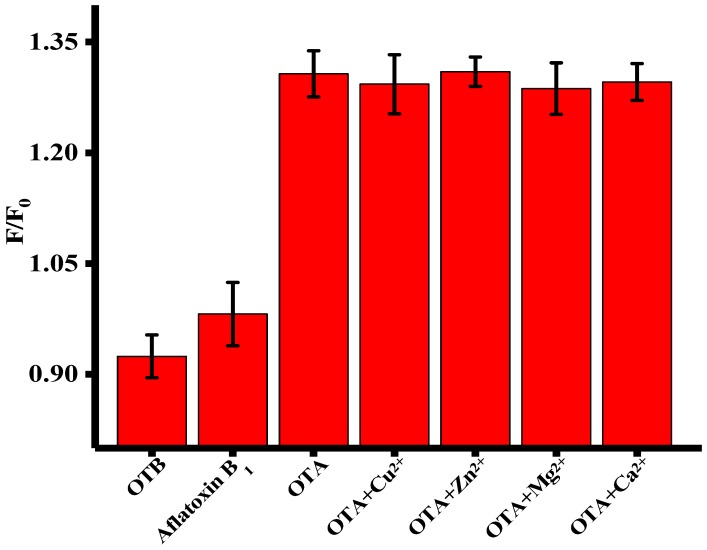
Selectivity of the proposed assay towards ochratoxin A (OTA), ochratoxin B (OTB), aflatoxin B_1_, OTA+Cu^2+^, OTA+Ca^2+^, OTA+Mg^2+^, OTA+Ca^2+^. F_0_, and F represent the fluorescence intensities in the absence and presence of OTA, respectively. Error bars are standard deviations of three experiments.

The fluorescence signals in response to the presences of various molecules, including OTA, OTB, and AFB_1_, were examined to evaluate the selectivity of the assay. As illustrated in Figure 4, the fluorescence signal ratio of OTA was much stronger than other toxins, due to the specific binding between OTA and OTA aptamer. In addition, a variety of possible metal cations in red wine were also studied. The occurrence of these metal cations does not interfere with fluorescent signals. This result demonstrate that the proposed method has excellent selectivity for OTA. The method was further applied to detect OTA in real samples.

### 2.6. Detection of OTA in Red Wine Samples

The practical application of the method was assessed by spiked recovery experiments using red wine samples. The results are shown in Table 2. The recoveries of OTA from the samples spiked with three different concentrations of OTA were varied from 92.2% to 111.6%. The data indicates that the detection of OTA in real samples with complex matrices by the proposed assay is feasible and reliable.

## 3. Conclusions

In summary, a novel and quencher-free fluorescence approach based on FAM-labeled OTA aptamer probe was developed for detecting OTA. The detection principle relies on the high sensitivity, specificity, and affinity between OTA and OTA aptamer, as well as the quenching ability of guanine induced by PIET. The proposed method had a linear concentration range from 3 to 300 nM, and a detection limit of 1.3 nM. Furthermore, the method exhibited excellent specificity towards OTA compared with that towards other toxins, and was successfully applied to detect OTA in red wine with the recovery rates of 93.8% to 111.6%. Accordingly, by using other appropriate aptamers, this approach may be applied to detect various other targets in other fields, such as the environmental and food safety fields.

## 4. Materials and Methods

### 4.1. Reagents

Ochratoxin A (OTA) and ochratoxin B (OTB) were obtained from Pribolab Co., Ltd. (QingDao, China). Aflatoxin B_1_ (AFB_1_) was purchased from Yuanye Co., Ltd. (Shanghai, China). All DNA probes (OTA aptamer and OTA-cAPT) were synthesized by Sangon Biotechnology Co., Ltd. (Shanghai, China). The sequence of DNA probes is shown in Table 3. The DNA sequence was dissolved using TE buffer and stored at −20 °C for further use. Tris[Tris-(hydroxy-methyl) aminomethane], magnesium chloride (MgCl_2_), hydrochloric acid (HCl), zinc chloride (ZnCl_2_), cupric sulfate (CuSO_4_), sodium chloride (NaCl), and calcium chloride (CaCl_2_) were purchased from Sinopharm Chemical Reagent Co., Ltd. (Shanghai, China). All reagents were of analytical-reagent grade and used without further purification. Ultrapure water (18.2 MΩ·cm) was prepared from a Milli-Q water purification system (Millipore Corp, Bedford, MA, USA) throughout the experiments.

### 4.2. Apparatus

The measurement of fluorescent intensity was carried out on an F-2700 fluorescence spectrophotometer (Hitachi, Tokyo, Japan). The measurement was conducted at room temperature with an excitation wavelength of 490 nm and wavelengths ranging from 505 to 600 nm. The slit widths for both excitation and emission were set to 10 nm.

### 4.3. Optimization of Reaction Conditions

To achieve optimum experimental results, several related reaction factors, including the concentration of OTA aptamer, reaction time for OTA aptamer and OTA-cAPT, reaction time for OTA, concentration ratio of OTA aptamer:OTA-cAPT, and length of OTA-cAPT, were optimized. The concentration of OTA aptamer was 200 nM, reaction time for OTA was 10 to 30 min, and that for the DNA probe (OTA aptamer/OTA-cAPT) was 5 to 60 min. The selected concentration ratios of OTA-aptamer:OTA-cAPT were 2:1, 1:1, 1:2, 1:3, and 1:4. The lengths of OTA-cAPT were 12, 15, 18, 21, and 24 nt. The concentration of OTA was 2 μM.

### 4.4. Detection of OTA by Fluorescence

Under the optimal conditions, different concentrations of OTA were mixed with 10 mM Tris-HCl (pH = 7.5), 120 mM NaCl, 20 mM CaCl_2_, 40 mM MgCl_2_, and 200 nM OTA aptamer and then incubated in a 37 °C water bath for 30 min. After that, 400 nM OTA-cAPT was added to a final volume of 100 μL at room temperature, and the reaction time was allowed to take place for additional 20 min. The fluorescence intensity was recorded at the wavelength range of 505 to 600 nm.

### 4.5. Selectivity Assay

In the experiment to verify the specificity of the probe, OTB and AFB_1_ were used as controls. Firstly, 300 nM each of OTA, OTB, AFB_1_, and OTA, containing 100 nM Cu^2+^, Zn^2+^, Mg^2+^, Ca^2+^ were added into the reaction solution containing 10 mM Tris-HCl (pH = 7.5), 120 mM NaCl, 20 mM CaCl_2_, 40 mM MgCl_2_, and 200 nM OTA aptamer and then incubated at 37 °C for 30 min. Subsequently, 400 nM OTA-cAPT was added to a final volume of 100 μL at room temperature. After 20 min of reaction, their fluorescence intensities were measured.

### 4.6. Assay of Real Samples

To test the feasibility of the method in the detection of OTA in real samples, red wine, which was bought from a local supermarket, was used as a real sample. The red wine samples were first filtrated to remove wine sediment and then diluted by 20 fold prior to subsequent analysis. In the spiked recovery experiment, a given amount of OTA was spiked into the diluted red wine samples, which was then subjected to subsequent sample handling and fluorescent measurement described above.

## Figures and Tables

**Table 1:** Comparison of different methods for OTA detection using aptasensors.

Method	LOD (nM)	Dynamic Range (nM)	Reference
Colorimetric	20	20–625	[33]
Colorimetric	10	10–200	[34]
Electrochemical	0.31	0.31–6.25	[13]
Fluorescent	9.8	10–100	[27]
Fluorescent	12.5	0–250	[35]
Fluorescent	2	2–200	[4]
Fluorescent	1.3	3–300	This work

**Table 2:** Recovery experiments of determination of ochratoxin A (OTA) in red wine samples.

Sample Number	Added (nM)	Detected (nM)	Recovery (%)
1	30	31.45 ± 2.35	104.8
2	80	75.05 ± 5.51	93.8
3	150	138.26 ± 1.84	92.2
4	200	223.14 ± 1.47	111.6

**Table 3:** Synthesized oligonucleotides (5′-3′) used in the experiment.

DNA Probe	Sequences (5′-3′)
OTA aptamer	GATCGGGTGTGGGTGGCGTAAAGGGAGCATCGGACA
cAPT12	CCA CAC CCG ATC GGGG
cAPT15	CACCCACACCCGATCGGGG
cAPT18	CGCCACCCACACCCGATCGGGG
cAPT21	TTACGCCACCCACACCCGATCGGGG
cAPT24	CCTTTACGCCACCCACACCCGATCGGGG
